# Clinical and molecular update of a large cohort followed by the Brazilian MODY multicenter study group (BRASMODY)

**DOI:** 10.1186/1758-5996-7-S1-A212

**Published:** 2015-11-11

**Authors:** Fernando de Mello Almada Giuffrida, Leticia Schwerz Weinert, Regina C S Moises, Luis Eduardo Calliari, Thais Della Manna, Lilian Araujo Caetano, Milena G Teles, Renata Pires Dotto, Ilda S Kunii, Sandra Pinho Silveiro, Magnus Regios Dias da Silva, André Fernandes Reis

**Affiliations:** 1 CEDEBA, Salvador, Brazil

## Background

Maturity-Onset Diabetes of the Young (MODY) is a group of monogenic forms of diabetes mellitus (DM) caused by mutations in at least 13 genes. Mutations in Glucokinase (GCK) and Hepatocyte nuclear factor-1 homeobox A (HNF1A) are the most common. Frequency of subtypes varies according to studied population. A broader view of MODY in Brazil is needed to improve diagnosis, epidemiological registry, and clinical care, also paving the way for future research.

## Objective

To update HNF1A and GCK mutations found in Brazilian patients with MODY phenotype, comparing its clinical characteristics to patients with a MODY phenotype but no mutations.

## Materials and methods

Literature review of published MODY mutations in Brazilian individuals was conducted. Unpublished clinical data of patients with MODY phenotype (both with/without mutations) were obtained directly from BRASMODY authors.

## Results

**Figure 1:**
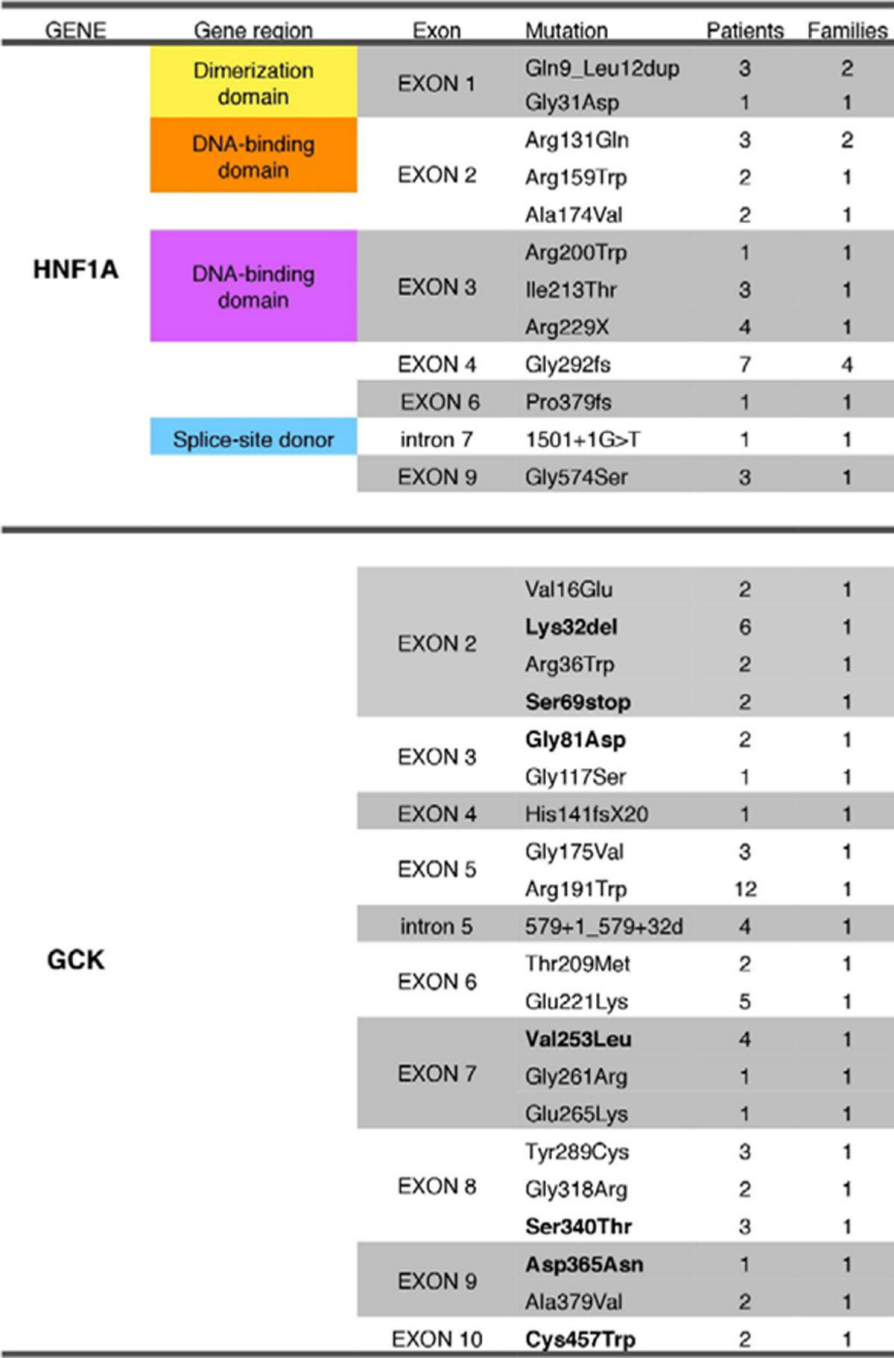
List of MODY mutations present in Brazilian individials (novel mutations in bold type).

285 Brazilian patients (119 families) have been enrolled. Twelve mutations in HNF1A and 21 in GCK were described in 92 individuals (Figure 1), 7 GCK mutations being first described in Brazilian subjects. Classical MODY criteria (early-onset familial DM) yielded low rates of GCK diagnosis (8.7%), whereas more specific criteria (non-progressive mild hyperglycemia) yielded close to 100% of diagnosis in recent studies. Classical criteria detected HNF1A mutations in only 20.2% of individuals. Comparing individuals with no mutations, GCK, and HNF1A, differences were seen in sulfonylurea use (31.8 vs. 7.3 vs. 58.3%, p 0.001), insulin use (45.3 vs. 4.9 vs. 16.7%, p < 0.001), hypertension (29.5 vs. 5.3 vs. 23.8%, p 0.009, all χ2, 2 df), and median C-peptide (1.11 vs. 1.60 vs. 0.93 ng/dL, p 0.049, Kruskal-Wallis). GCK patients had lower age at diagnosis (17.9±13.9 vs. 26.7±13.0 yrs., p 0.001), body mass index at diagnosis (19.2±4.5 vs. 24.7±3.7 kg/m2, p 0.006), and HbA1c (6.32±0.54 vs. 7.77±2.66, p 0.008, all ANOVA/Tukey) than individuals without mutations. No phenotype/genotype correlation was observed in both GCK and HNF1A groups.

## Conclusions

MODY mutations in Brazilian individuals show clinical distinction among different subtypes of monogenic DM, mainly between GCK and other MODY types. Mild non-progressive hyperglycemia was an adequate screening tool for GCK mutations. Given the low positivity rate of HNF1A mutations, new recruitment strategies are needed. Although rare, other monogenic forms of DM types should be investigated in this subgroup of patients without GCK/HNF1A mutations.

